# Supplementary material for: Extending Integrate-and-Fire Model Neurons to Account for the Effects of Weak Electric Fields and Input Filtering Mediated by the Dendrite
Source: PLoS Comput Biol. 2016 Nov 28;12(11):e1005206. doi: 10.1371/journal.pcbi.1005206 (PMC5125569; doi:10.1371/journal.pcbi.1005206)
Supplement: S1 Text — Fig A: Reproduction of spiking activity for dendritic inputs using EIF type models. Fig B: Spike rate modulation due to an electric field for somatic inputs using neuron models of the EIF type. Fig C: Spike rate modulation due to an electric field for distal dendritic inputs using neuron models of the EIF type. (PDF) [file pcbi.1005206.s001.pdf]

— Supplementary material —

Extending integrate-and-fire model neurons to account for the effects of weak electric fields and input filtering mediated by the dendrite

Florian Aspart[1,2,*,‡], Josef Ladenbauer[1,2,*,‡], Klaus Obermayer[1,2,‡]

1 Department of Software Engineering and Theoretical Computer Science, Technische Universität Berlin, Germany
2 Bernstein Center for Computational Neuroscience Berlin, Germany

∗ **These authors contributed equally to this work**
‡ **E-mail: florian.aspart@tu-berlin.de, josef.ladenbauer@tu-berlin.de, klaus.obermayer@tu-berlin.de**

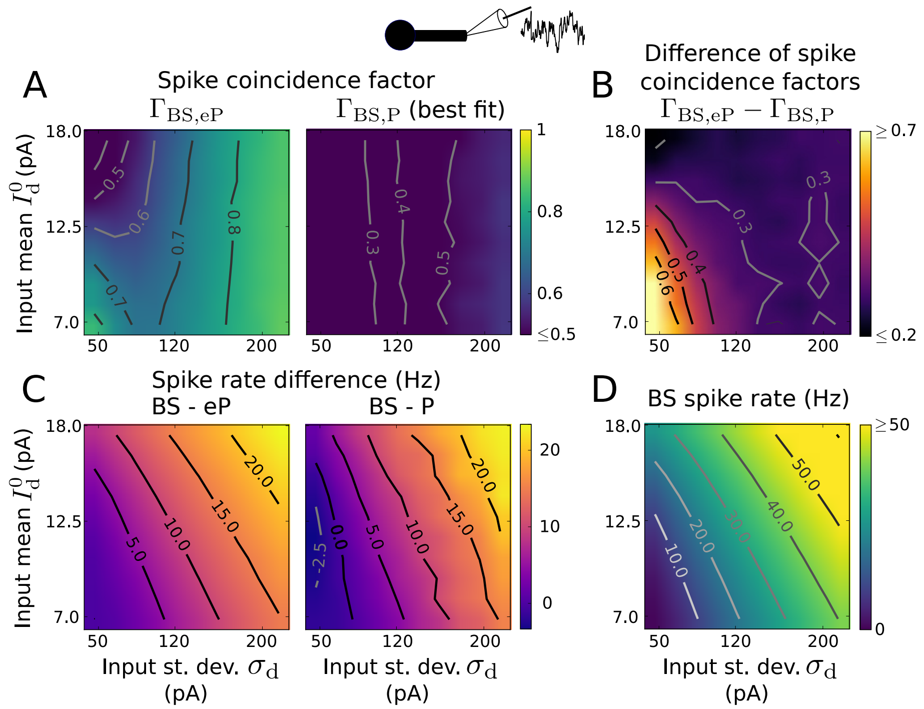

Supplementary Figure A. Reproduction of spiking activity for dendritic inputs using EIF type models A: Coincidence factor for the BS and eP model spike trains, $\Gamma_{\mathrm{BS,eP}}$ (left), and for the BS and P model spike trains, $\Gamma_{\mathrm{BS,P}}$ (left) as a function of input mean I_{d}^0 and standard deviation σ_{d}. The parameter values of the P model were optimized to maximize $\Gamma_{\mathrm{BS,P}}$ for each input (i.e., (I_{d}^0, σ_{d})-pair) independently. B: Difference $\Gamma_{\mathrm{BS,eP}} - \Gamma_{\mathrm{BS,P}}$ between the coincidence factors shown in B. C: Spike rate difference of the BS and eP models (left) and of the BS and P models (right) as a function of I_{d}^0 and σ_{d}. D: Spike rate of the BS neuron model. Results presented in A-D show averages over 6 noise realizations. The parameter values of the BS model are listed in Table 1.

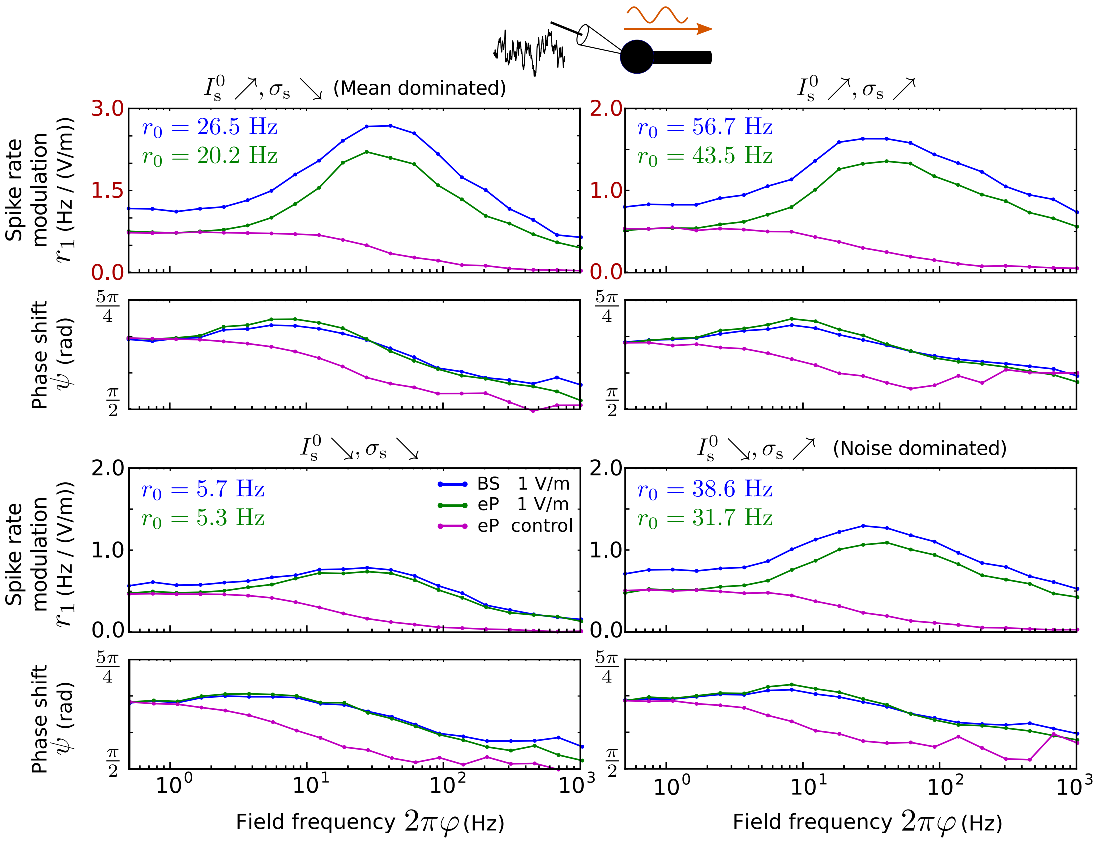

Supplementary Figure B. Spike rate modulation due to an electric field for somatic inputs using neuron models of the EIF type Spike rate modulation of the BS (blue) and the eP (green) models due to an oscillating electric field ($E_1 = 1$ V/m) as a function of its frequency, for different somatic inputs: $I_s^0 = 10.61$ pA, $\sigma_s = 24.08$ pA (top left), $I_s^0 = 10.61$ pA, $\sigma_s = 68.21$ pA (top right), $I_s^0 = 5.05$ pA, $\sigma_s = 24.08$ pA (bottom left), and $I_s^0 = 5.05$ pA, $\sigma_s = 68.21$ pA (bottom right). Magenta lines show the spike rate modulation of the eP model for which I_E was given by $I_E(t) = I_1 \sin(\varphi t + \phi)$ with constant amplitude $I_1 = |B(0.5/(2\pi))|$ and phase shift $\phi = \arg(B(0.5/(2\pi)))$ with B from Eq. 21 and $E_1 = 10$ V/m. Note the different amplitude scales in the two top plots.

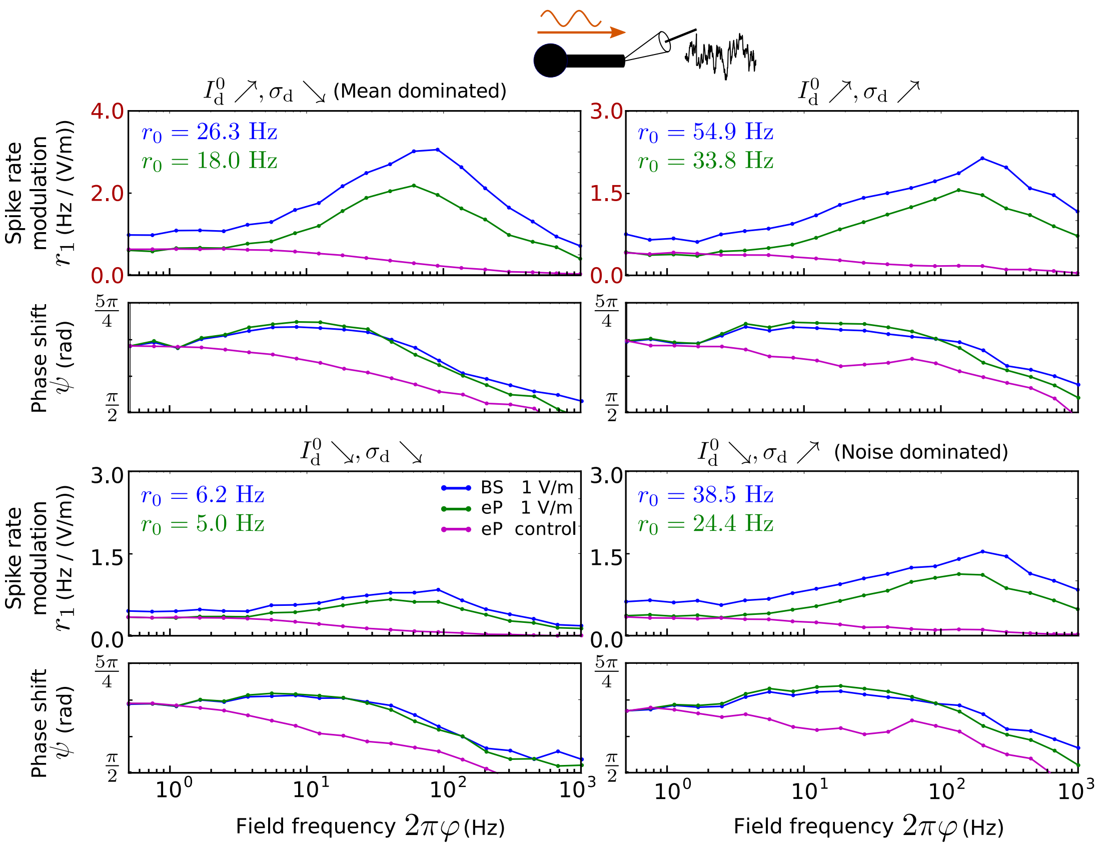

Supplementary Figure C. Spike rate modulation due to an electric field for distal dendritic inputs using neuron models of the EIF type Spike rate modulation of the BS (blue) and the eP (green) models due to an oscillating electric field ($E_1 = 1$ V/m) as a function of its frequency, for different distal dendritic inputs: $I_\mathrm{d}^0 = 16.73$ pA, $\sigma_\mathrm{d} = 57.73$ pA (top left), $I_\mathrm{d}^0 = 16.73$ pA, $\sigma_\mathrm{d} = 203.41$ pA (top right), $I_\mathrm{d}^0 = 7.56$ pA, $\sigma_\mathrm{d} = 57.73$ pA (bottom left), and $I_\mathrm{d}^0 = 7.56$ pA, $\sigma_\mathrm{d} = 203.41$ pA (bottom right). Magenta lines show the spike rate modulation of the eP model for which I_E was given by $I_E(t) = I_1 \sin(\varphi t + \phi)$ with constant amplitude $I_1 = |B(0.5/(2\pi))|$ and phase shift $\phi = \arg(B(0.5/(2\pi)))$ with B from Eq. 21 and $E_1 = 10$ V/m. Note the different amplitude scales in the two top plots.
